# Unveiling malaria vectors: species complex and surveillance insights from Sundergarh, Odisha, India

**DOI:** 10.1186/s41182-025-00719-3

**Published:** 2025-04-07

**Authors:** Taru Singh, Syed Shah Areeb Hussain, K. Pradhan, Monica Rawat, Ramesh Chand Dhiman

**Affiliations:** 1 Environmental Epidemiology Division, National Institute of Malaria Research- Indian Council of Medical Research, Dwarka Sector 8, New Delhi, 110077 India; 2 Centre for Medical Biotechnology, Amity Institute of Biotechnology, Amity University, Sector 125, Noida, Uttar Pradesh 201313 India; 3 Odisha Field Unit, National Institute of Malaria Research- Indian Council of Medical Research, Dwarka Sector 8, New Delhi, 110077 India

**Keywords:** Sibling species, *Anopheles culicifacies*, Polymerase Chain Reaction, Entomological surveillance, Odisha

## Abstract

**Background:**

Malaria is one of the most infectious life-threatening vector-borne diseases affected by climate change. Because of the emerging climate change problem, it was thought prudent to identify prevalent mosquito species and find the malaria parasite's presence in field-collected mosquitoes in Odisha.

**Material and methods:**

The study was undertaken at four villages in the Rourkela, Sundergarh district of Odisha, India, from January 2018 to January 2020, generating entomological and climatic data. Field-collected mosquitoes were processed, and DNA was extracted, followed by multiplex PCR for differentiation of sibling species for *Anopheles culicifacies* and *Anopheles fluviatilis* mosquitoes. Enzyme-Linked immunosorbent assay was also performed for detection of circumsporozoite proteins of Plasmodium. Sequencing was performed, and a phylogenetic tree was constructed using the neighbor-joining method.

**Results:**

For *Anopheles culicifacies*, 43.25% of mosquitoes belonged to sibling species C, followed by species B, A, and D. Similarly, for *Anopheles fluviatilis*, sibling species T was found in 57.5%, followed by species U and S. Sibling species were confirmed on the difference in the sequences of conserved regions of the 28S rDNA.

**Conclusions:**

We can conclude that sibling species C (*Anopheles culicifacies*) was predominant in Rourkela, and sequencing further confirmed the presence of parasites (*Plasmodium vivax*) in *Anopheles culicifacies* as sibling species C.

## Introduction

Malaria is a major public health problem, with an estimated 263 million cases worldwide in 2023, of which India accounted for a significant proportion. The estimated number of malaria deaths stood at 597,000 globally (WHO, 2024) [1]. Between 2022 and 2023, India has reduced its malaria burden significantly, with almost a 9.6% reduction in cases reported [1]. The state of Odisha has been at the forefront of this crusade against malaria and has achieved a reduction of 94% in malaria cases only in the past five years. However, the closer we get to the goal of elimination, the slower the rate of reduction in malaria incidence, making it more and more difficult to eliminate malaria [2].

One of the crucial tools in this fight against malaria has been Indoor Residual Spraying (IRS) conducted at least two or three times a year in malaria endemic regions. However, for effective utilization of IRS, it is essential to identify the types of vectors, their role in transmission, and the months of peak abundance for fine-tuning IRS timing. Malaria transmission in Odisha is highly complicated, owing to the presence of two primary vectors of malaria, *Anopheles culicifacies* and *Anopheles fluviatilis* [3], as well as several secondary vectors, such as *Anopheles sub-pictus, Anopheles annularis*, and *Anopheles varuna* are found in the majority [4]. The various topographical features present in Odisha, such as fertile coastal plains, mountains, highlands, and forests, support different types of vector populations, leading to differences in malaria transmission. Therefore, a holistic understanding of the interplay between these vectors and their preferred habitats is crucial for the effective implementation of IRS and elimination strategy in Odisha.

*An. culicifacies* is the primary rural/peri-urban vector of malaria in India and was responsible for the appearance of malaria in India and other Southeast Asian countries [5, 6]. Based on the differences in the polytene chromosome, five sibling species of the *An. culicifacies* have been identified, i.e. A, B, C, D, and E, with differences in their bionomics and role in malaria transmission [7]. All members of *An. culicifacies* complex except E have been found to be predominantly zoophilic [8]. In the hills and foothills of Odisha, *An. fluviatilis* is the primary malaria vector [9], which breeds in slow-running water. Based on differences in distribution pattern, feeding preferences, disease transmission, and genotypes present on the polytene chromosome, the *An. fluviatilis* complex has been classified into sibling species S, T, and U [10]. Species S is regarded as a highly efficient vector of malaria, unlike species T and U, which are considered non-vectors [10].

Malaria transmission is a complex process influenced by environmental, ecological, and biological factors, with climate change and sibling species diversity playing key roles. Rising global temperatures, changing rainfall patterns, and increased humidity are expanding the range of malaria vectors like *Anopheles culicifacies*, altering disease spread and prevalence [11]. Sundergarh, Odisha, a malaria-endemic region, presents a unique challenge for disease control, where accurate vector identification is essential. The presence of closely related sibling species complicates control efforts, as they differ in insecticide resistance, feeding habits, and ability to transmit malaria [12, 13].

Molecular surveillance has become a crucial tool for identifying sibling species, allowing for more effective and targeted interventions [14]. Climate change is further impacting vector survival, breeding patterns, and malaria transmission dynamics. Warmer temperatures have enabled *Anopheles culicifacies* to spread into higher altitudes and previously malaria-free areas [11]. Increased rainfall and humidity create ideal breeding conditions, leading to frequent malaria outbreaks [15]. Differences in vectorial capacity among sibling species mean that some play a greater role in disease transmission than others [12]. Moreover, variations in insecticide resistance among these species pose challenges for vector control [13]. To improve malaria control in regions like Sundergarh, integrating molecular surveillance with climate-based predictive models is essential. This approach can help track vector population dynamics and insecticide resistance patterns and guide targeted interventions.

In this study, the predominance of the two vector species and their role in malaria transmission in two different ecotypes, namely forested and riverine, in the Sundargarh district of Odisha were assessed. The study assesses how the climate in these two ecotypes is related to the prevalence of different vector species of malaria and its role in malaria transmission in the region. The results of the study would help guide the national program in fine-tuning IRS strategy based on the vector to target as well as their relative abundance across different months.

## Results

### Climate of forested and riverine villages

**Fig. 1:**
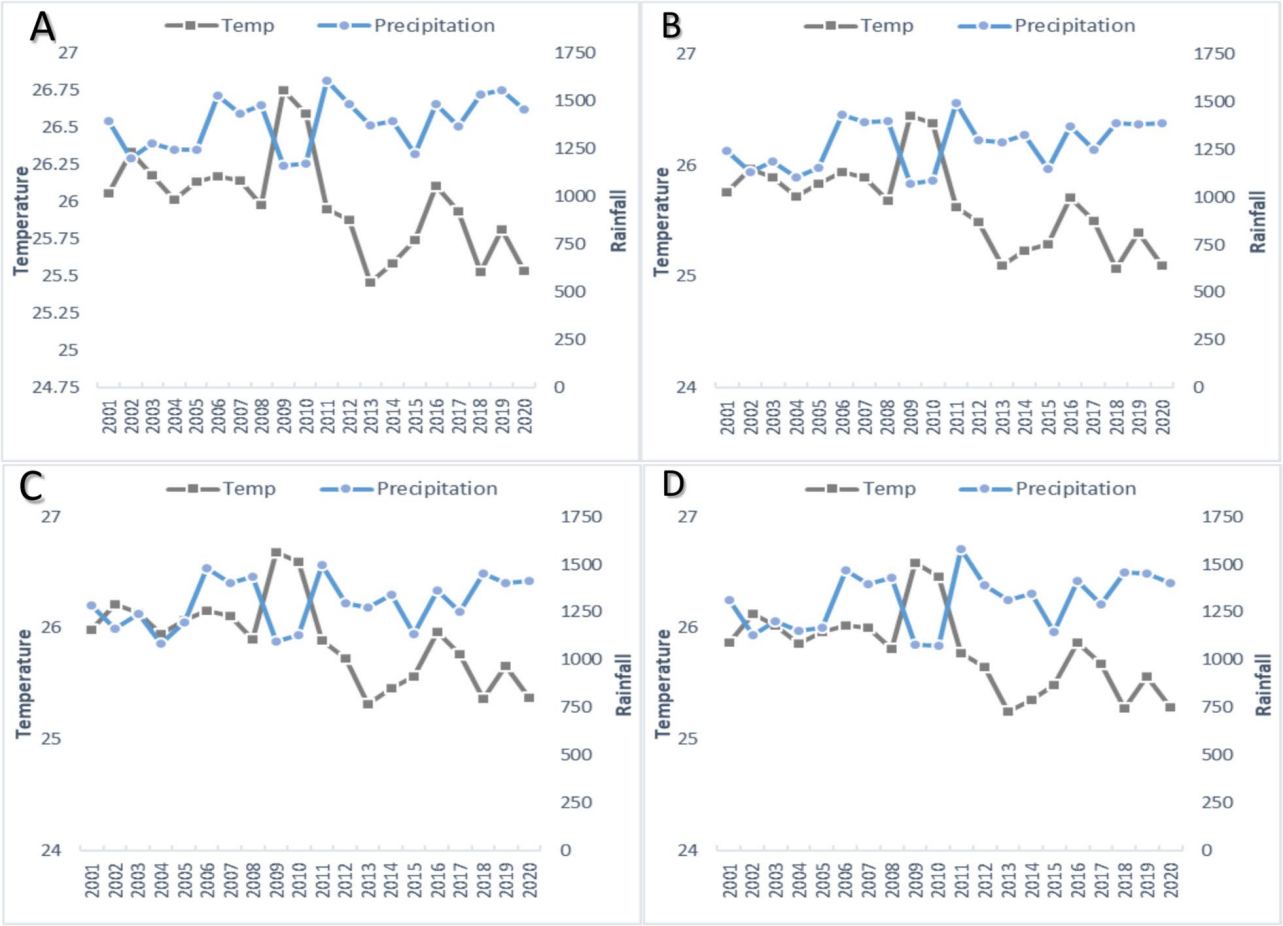
Average annual and temperatures since 2001–2020 in the four study villages **a** Mahaliyapalli, **b** Nuagaon, **c** Benuam and **d** Rangamati (Data obtained from [16])

Average annual temperatures in the four study sites range between 25 and 27 ⁰C, whereas total annual rainfall ranges from 1073 to 1608 mm. The climatic profile of the district and the four study sites shows that Sundargarh has a strong propensity for malaria transmission with ambient temperatures for most of the year and ample rainfall to support breeding. Long-term trends (2001–2020) in temperature and precipitation patterns show that over the period of 20 years, the average annual temperature in all four villages has reduced, whereas total annual rainfall has increased marginally (Fig. 1) [16]. This has likely made both forested and riverine villages more suitable for malaria transmission.

### Man hour density (MHD)

MHD of collected mosquitoes is shown in Table 1. Among thirteen different anopheline species in Odisha, *An. culicifacies* and *An. fluviatilis* were the known primary vectors. MHD of *An. culicifacies* was found highest in all four villages.

**Table 1:** Correlation of MHD of *An. culicifacies* and *An. fluviatilis* with temperature and rainfall in the four study villages of Sundargarh, Odisha

Village	Mosquito species	Temperature	Rainfall
Mahaliyapalli	MHD (*An. culicifacies*)	− 0.680 *(0.000)*	− 0.585 *(0.003)*
	MHD (*An. fluviatilis*)	–	–
Nuagaon	MHD (*An. culicifacies*)	0.187 *(0.372)*	0.633 *(0.001)*
	MHD (*An. fluviatilis*)	–	*–*
Benuam	MHD (*An. culicifacies*)	0.352 *(0.084)*	0.546 *(0.005)*
	MHD (*An. fluviatilis*)	− 0.412 *(0.040)*	− 0.233 *(0.262)*
Rangamati	MHD (*An. culicifacies*)	0.076 *(0.716)*	0.555 *(0.004)*
	MHD (*An. fluviatilis*)	− 0.428*(0.033)*	− 0.038 *(0.855)*

**Fig. 2:**
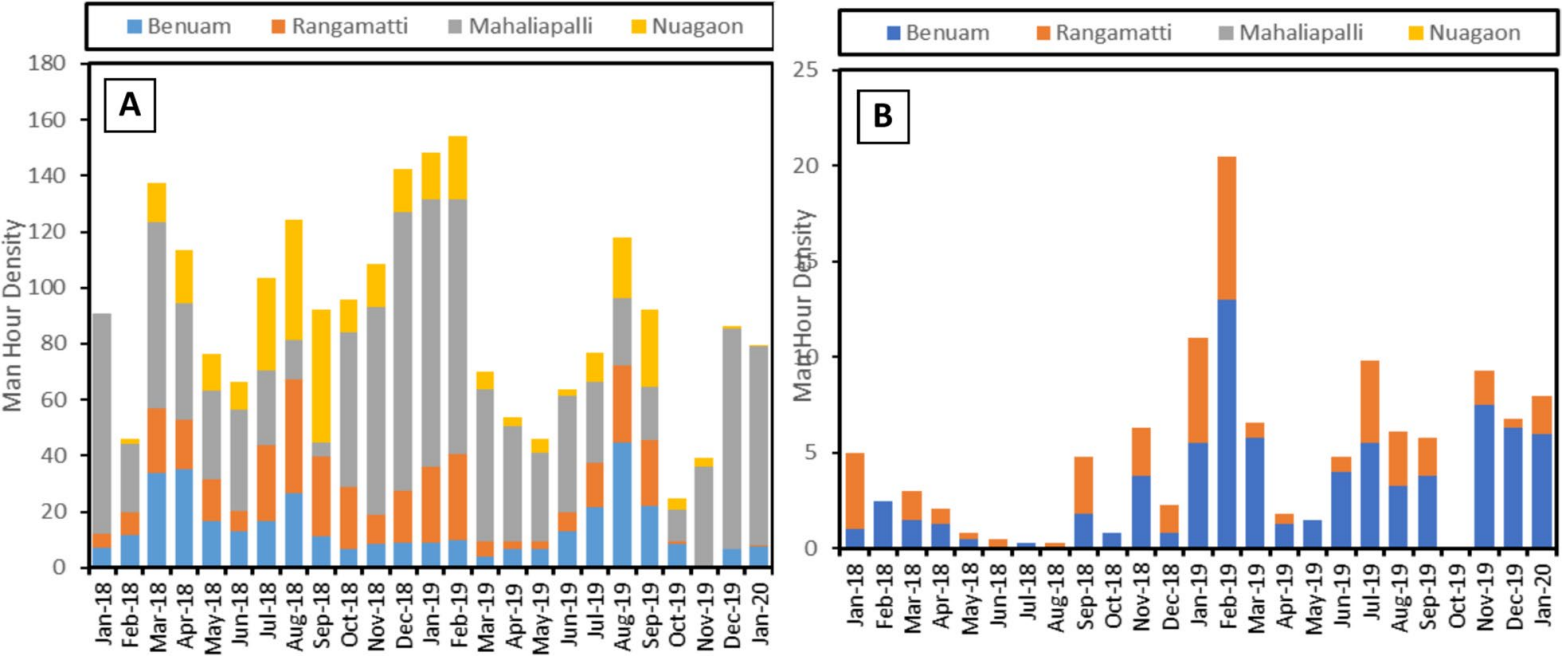
Relative prevalence of **a** *An. culicifacies* and **b** *An. fluviatilis* in the selected villages of Sundergaragh district, Odisha

*An. culicifacies* was the most predominant vector species (93.5%) in all four villages. *An. fluviatilis* was mainly found in forested villages Benuam and Rangamatti, although with a lower MHD (0–13) than *An. culicifacies* (2.8 to 44.8). In riverine villages, only a couple of adult *An. fluviatilis* were collected, mainly during the winter season, and MHD was 0 for most parts of the year. However, *An. culicifacies* were abundant with MHD ranging from 0.5 to 99.5. Culex mosquitoes were also collected in large numbers. In both forested and riverine villages, mosquitoes were mostly prevalent in cattle sheds (~ 90%), where the man-hour density (MHD) ranged from 12 to 192, and only a small number of mosquitoes were found in human dwellings (MHD 0–10) Fig. 2**.**

### Relation between vector prevalence and climatic factors

To assess how the monthly climate affected the distribution of the two vectors, *An. culicifacies* and *An. fluviatilis*, the monthly temperature and rainfall over the whole study period were plotted against the man-hour density of the mosquitoes collected (Fig. 2). Monthly temperatures in the four villages range between 17 and 34 ⁰C, whereas rainfall ranges from 0.9 to 624 mm. The riverine villages, particularly Mahaliyapalli, received more rainfall than the forested period over the study time. Temperatures remain above 20 ⁰C for most of the year and drop below 18 ⁰C only in the months of December and January, which significantly lowers the vector population along with the malaria incidence.

**Fig. 3:**
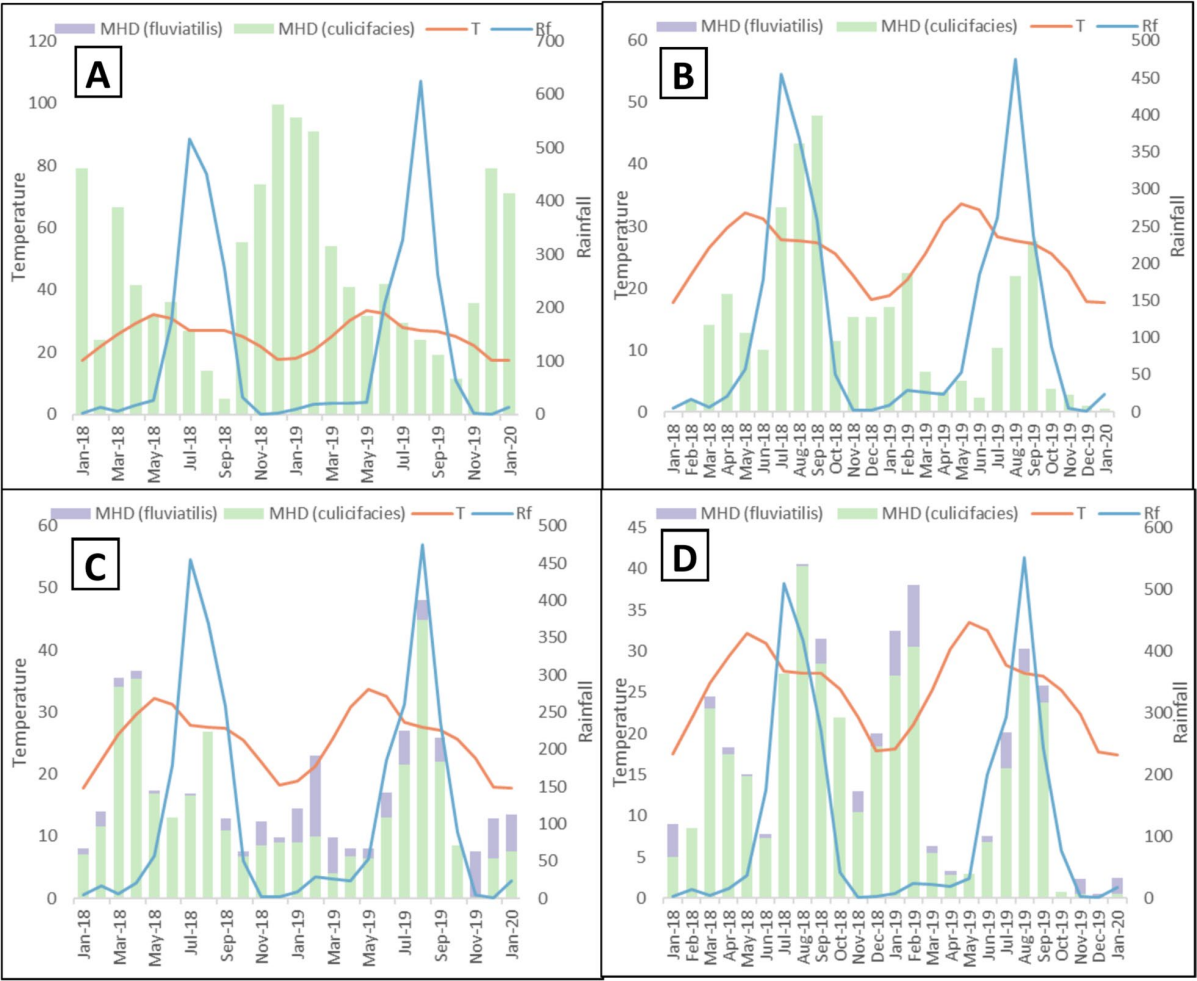
Average monthly temperature (T), rainfall (Rf) and MHD of the two primary vectors *An. culicifacies* and *An. fluviatilis* in the Riverine villages **a** Mahaliyapalli and **b** Nuagaon; as well as the forested villages **c** Benuam and **d** Rangamati

Pearson’s correlation test was also conducted to assess the impact of temperature and rainfall on the density and prevalence of the two main Anopheline vectors in Odisha. *An. culicifacies* and *An. fluviatilis.* In riverine villages (Mahaliyapalli and Nuagaon), *An. fluviatilis* was found absent, and both temperature and rainfall were found to show significant correlation with the density of *An. culicifacies* (Table 1) in Mahaliyapalli (-0.68 and -0.585, respectively, p < 0.003), whereas only rainfall showed significant correlation with *An. culicifacies* density in Nuagon (0.633, p = 0.001)*.* On the other hand, in the forested villages (Benuam and Rangamati), both vectors were present, and it was found that, while rainfall affected the prevalence of *An. culicifacies* (0.546, p = 0.005), temperature was the limiting factor for the prevalence of *An. fluviatilis* (-0.428, p = 0.033) (Table 1) (Fig. 3).

### Identification of vector sibling species through Multi-plex PCR

**Fig. 4:**
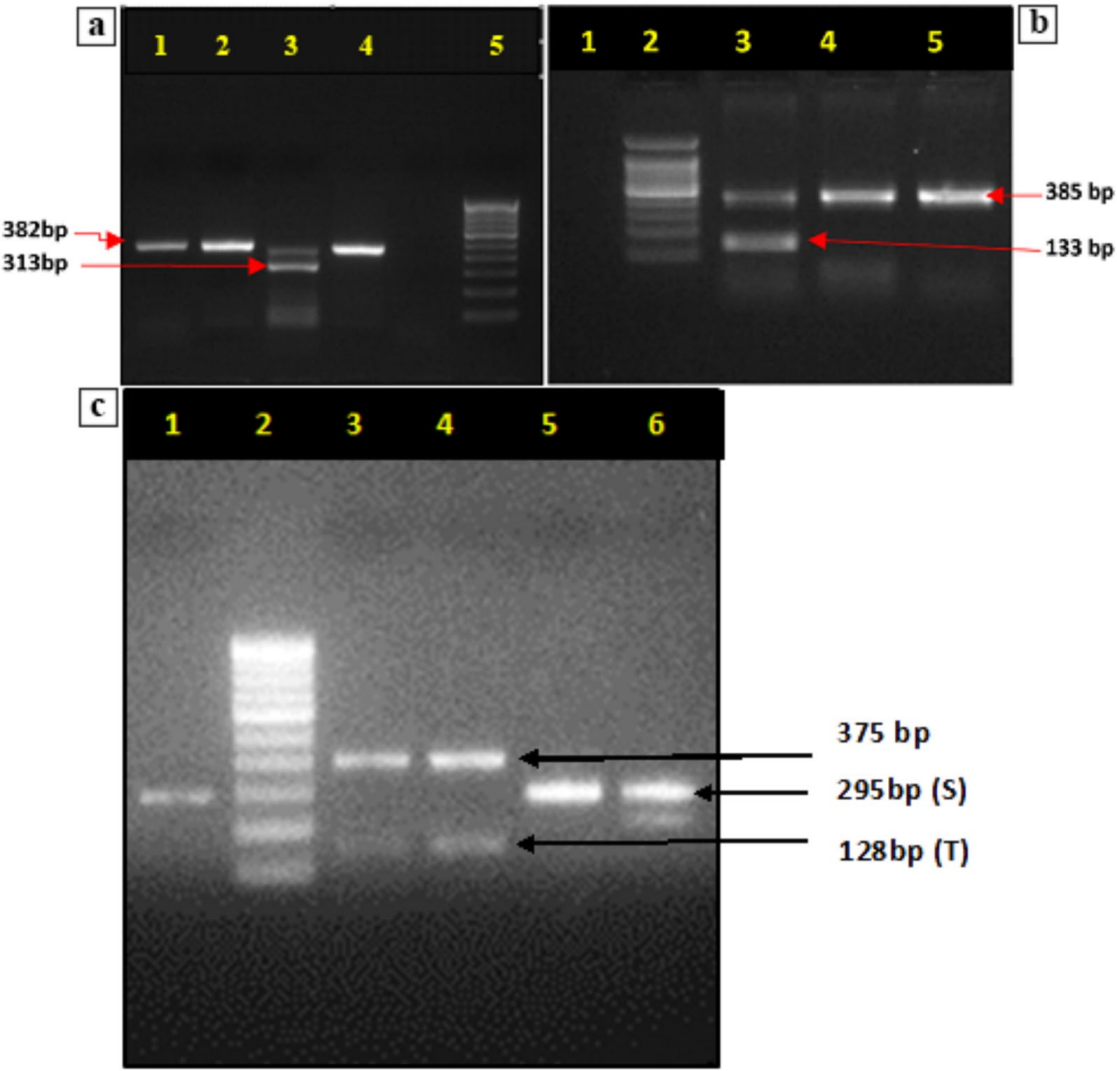
Multiplex PCR for *An. culicifacies* and *An. fluviatilis* sibling specific genes. **a** *An. culicifacies:* Lane 1,2 & 4: A/D species common product (382 bp), Lane 3: A/D species-specific product (313 bp), Lane 5: negative control, and Lane 6: 100 bp ladder. **b** *An. culicifacies:* Lane 1: negative control, Lane 2: 100 bp ladder, Lane 3: B/C/E species common product (133 bp & 385 bp) and Lane 4 & 5: B/C/E specific product (385 bp). **c** *An. fluviatilis:* Lane 1, 5 & 6: Sibling species S (295 bp), Lane 2: ladder (100 bp), Lane 4: Sibling species T (128 bp) and Lane3: Sibling species U (375 bp)

1765 mosquitoes (1485 *An. culicifacies* and 280 *An. fluviatilis*) were screened by allele-specific PCR assay to identify sibling species. Three primers, i.e., D3A/D3B (universal primers) ACA/ACB, and AFS/AFT, were used in multiple combinations to identify three sibling species of *An. fluviatilis.* Analysis revealed a predominance (*An. culicifacies*) of sibling species C (43.39%) followed by sibling species B (36.29%), A (14.68%), and D (5.72%). For *An. fluviatilis* sibling species, T was predominant (57.5%), followed by species U (27.5%) and S (17.14%). A representative photograph of the gel is shown in Fig. 4**.**

### Detection of Malaria parasites in vectors using ELISA

ELISA confirmed the presence of the parasite (*P. vivax*) in 18 *An. culicifacies* mosquitoes collected from the cattle shed of Mahayapalli, a riverine village, during September. No absorbance was observed in the remaining 4135 samples. A visual reading of the ELISA plate at 405–414 nm revealed the absorbance for each well of the ELISA plate except for two wells for which OD was like that of the positive control, indicating the absence of circumsporozoite antigen.

### Sequencing analysis

**Fig. 5:**
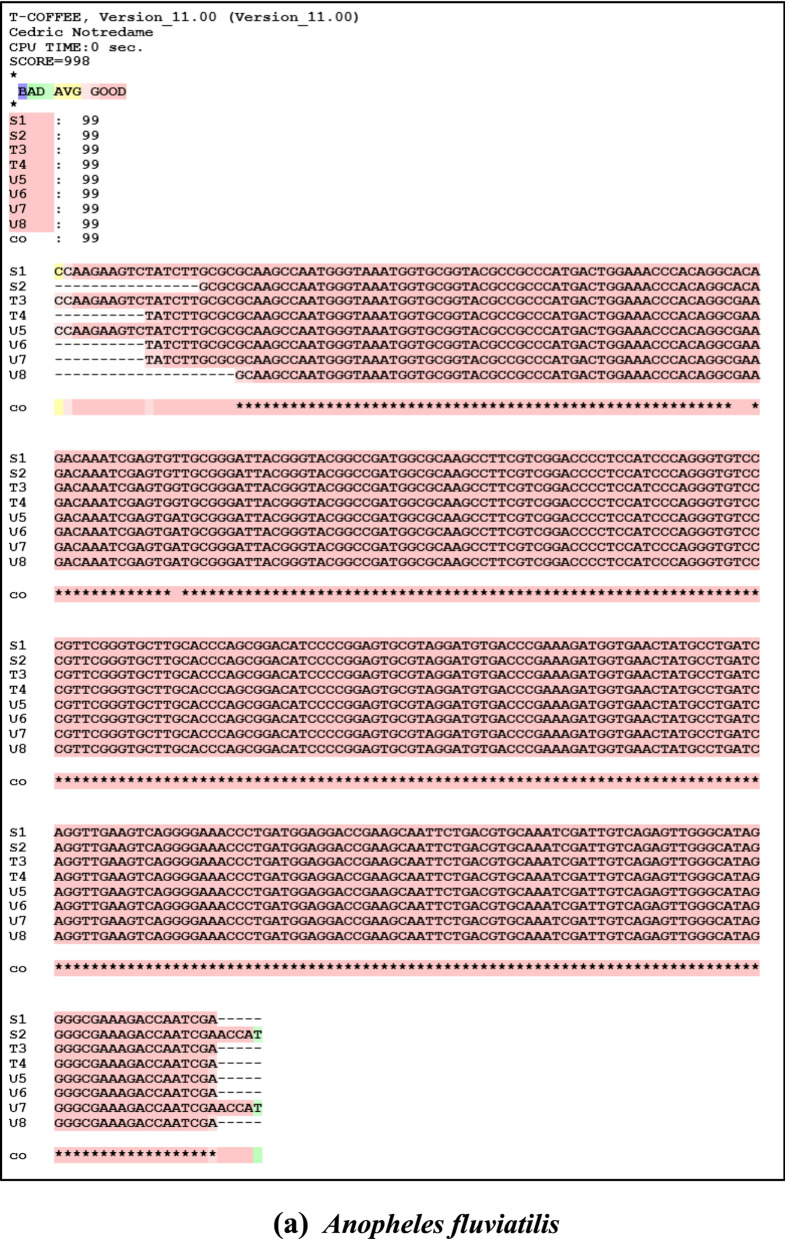

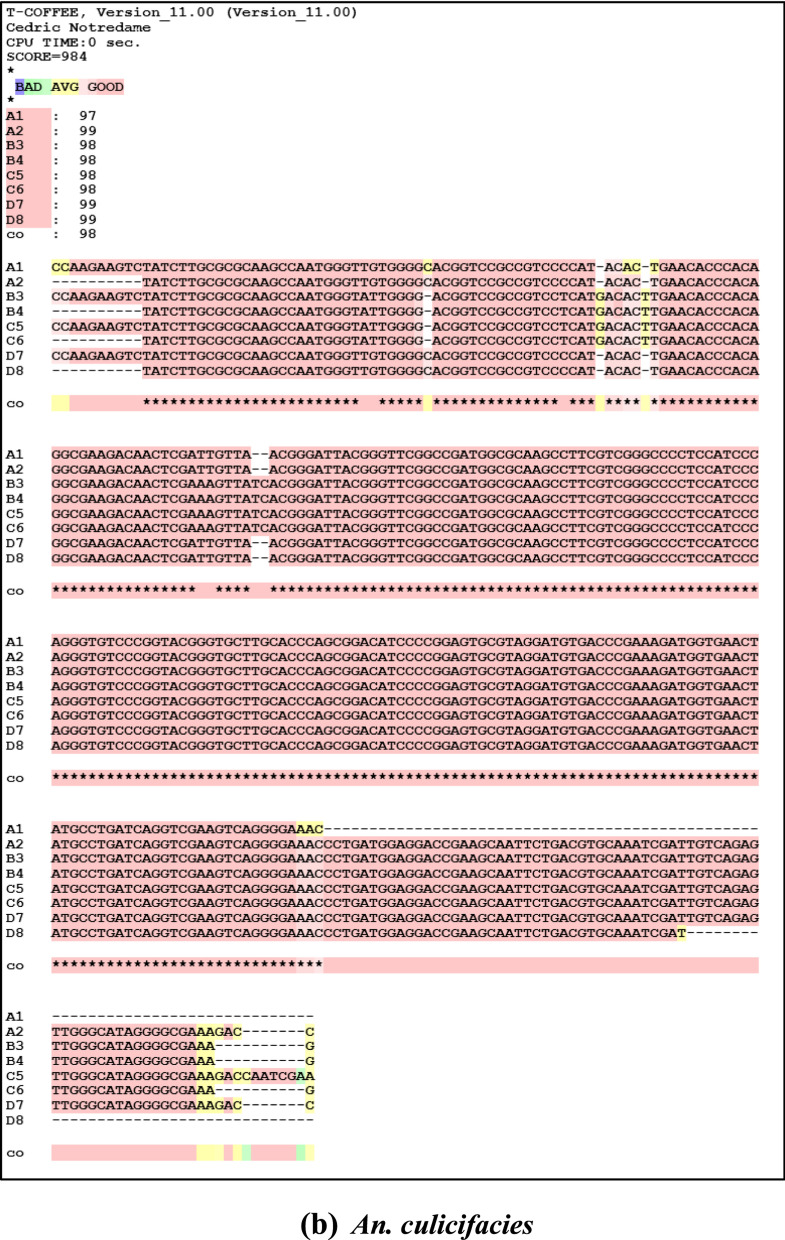
Multiple Sequence alignment of **a** *An. fluviatilis* and **b** *An. culicifacies* sibling species using T-COFFEE (version 11.00). Sequence S1 and S2 correspond to sibling species S, sequences T3 and T4 belong to sibling species T, and sequences U5, U6, U7, and U8 correspond to sibling species U of *An. fluviatilis*. Similarly, for *An. culicifacies* sequence A1, A2 corresponds to sibling species A, sequence B3, B4 corresponds to sibling species B, sequence C5, C6 corresponds to sibling species C, and sequence D7, D8 corresponds to sibling species D

Sequence analysis was done after performing BLAST and multiple alignments. Nucleotide accession numbers were obtained from NCBI as follows: for *An. fluviatilis* **MK281592**, **MK281593** for species S, **MK281595** for species T, and **MK281598**, **MK281599** for species U; for *An. culicifacies* **MK377005**, **MK377006** for species A, **MK377007**, **MK377008** for species B, **MK377009**, **MK377010** for species C, and **MK377011** and **MK377012** for species D. Sequences of sibling species *An. fluviatilis* and *An. culicifacies* are different at sixteen positions, i.e. 38 (T-G), 41 (T-G), 57 (A-C), 68 (G-A), 70 (A-C), 92 (A-G), 97 (T-A), 94 (C-A), 95 (G-A), 96 (A-G), 97 (G-T), 99 (G-A), 101 (T-C), 102 (G-A), 116 (A-T) and 143 (A-G) respectively. Sibling species of *An. culicifacies* A/D are different from sibling species B/C at positions 43 (C-A), 44 (G-C), 46 (T-G), 49 (G-C), 50 (C-G), and 52 (G-C). While sibling species S of *An. fluviatilis* have nucleotide “A’ in place of nucleotide “G’’ at position 81 in species U and T and all the three sibling species S (T), T (G) and U (A) are different at position 100 (Fig. 5a and b).

### Phylogenetic analysis

**Fig. 6:**
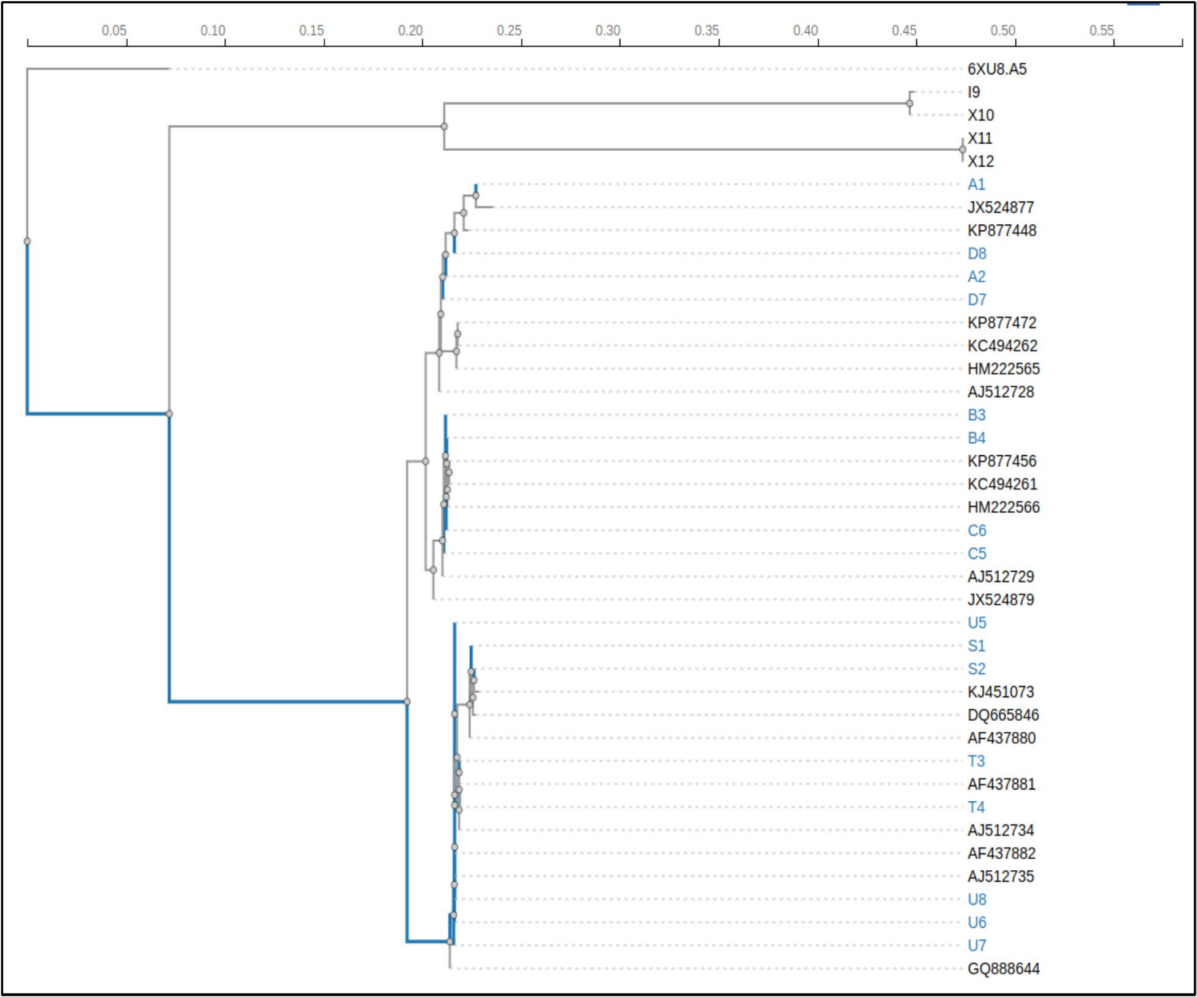
Phylogenetic tree illustrating the evolutionary relationships among various sibling species sequences of *An. culicifacies* and *An. fluviatilis*, [with 6XU8.A5 (*ribosomal sequence of Drosophila melanogaster*) used as the outgroup to root the tree]. Bootstrap values are shown at the nodes, indicating the confidence level of the branching points. The tree was constructed using the MEGA11 software, providing insights into the genetic divergence and grouping of the sequences based on their evolutionary history

This study constructed a phylogenetic tree using sixteen 28S rRNA sequences (Fig. 6). It can be readily evident from the tree that it was categorized into five subgroups based on the percentage of similarity (98%) and dissimilarity (2%) among sequences.

## Discussion

Odisha is one of the states with very high malaria endemicity in India. While recent years have experienced a significant reduction in the number of malaria cases in the state, further progress towards the elimination of malaria will require a sound understanding of the local vector bionomics and their interaction with climatic and ecological factors. These factors have contributed to highly variable vector distribution in different parts of the state. They are responsible for the wide disparity in the endemicity of malaria in other parts of Odisha. Local and focal factors are also responsible for malaria transmission, depending on the vector abundance under favorable conditions [3].

Vector abundance varies significantly in Odisha over the year, with two significant peaks, once in the monsoon months and the other in the winter months, when temperatures are between 20 ℃ and 30 ℃. *An. culicifacies* showed higher prevalence in both riverine and forested villages during the monsoon months, with a smaller peak during January and February. In contrast, *An. fluviatilis* abundance peaked during the winter months, particularly in February, with a smaller peak in the monsoon months of July and August. This difference in the monthly prevalence of the two species has been observed in previous studies as well [17, 18] and may be related to the differences in temperature tolerance in different vector species.

Odisha experiences a hot and humid climate, and trees in forested areas provide adequate tree canopy and respite to the mosquitoes from the heat as mosquitoes take shelter in tree holes [19, 20]**.** This could be why higher MHD was observed in the forested villages as compared to the riverine villages. As Odisha becomes hotter because of climate change, vector species may retreat to the forested areas in the future, where the climate is balanced. In concurrence with the present understanding of vector distribution, riverine villages were dominated by *An. culicifacies,* whereas forested villages were more suitable for *An. fluviatilis* with some presence of *An. culicifacies.*

There is a need to understand species-specific bionomics of the prevalent vector species to take preventive measures accordingly. Our study showed the prevalence of *An. culicifacies* sibling species C (43.25%) in field-collected mosquitoes, followed by species B, A, and D. This pattern of sibling species has also been observed in other studies in Odisha [10, 21]. For *An. fluviatilis* sibling species, T was the most prevalent (57.5%), followed by species, U and S. Previous studies show some disparity in the prevalence of different sibling species of *An. fluviatilis*, and while [22] found a higher majority of sibling species T, [10] found that sibling species S were more abundant. Both *An. culicifiacies* sibling species C and *An. fluviatilis* sibling species T are primarily zoophilic, and most of the adult vectors collected were found in cattle sheds rather than in human dwellings. The difference in the occurrence of different sibling species of two vectors may be due to the difference in malaria burden and transmission rate in Odisha because of its environmental diversity. ELISA also showed a low sporozoite rate of 0.43% in *An. culicifacies* mosquitos for *P. vivax*. The low sporozoite rate in the prevalent vector species in Odisha can be attributed to a combination of factors related to the behaviour and ecology of the mosquitoes. One key factor is the blood meal preference of these mosquitoes. Studies indicate that certain Anopheles species in the region, including those in nearby areas, exhibit a preference for feeding on animals rather than humans. This zoophilic behaviour reduces the likelihood of mosquitoes picking up and transmitting the malaria parasite from human hosts, thereby lowering the overall sporozoite rate.

For instance, research has shown that mosquitoes in Odisha often prefer to feed on cattle, which diverts their contact away from humans and interrupts the transmission cycle of Plasmodium parasites. Additionally, the proximity of cattle sheds to human dwellings means that even though humans are nearby, the mosquitoes still choose animal blood meals due to easier access, which further diminishes the chances of malaria transmission.

Furthermore, the resting behaviour of mosquitoes also plays a role. Mosquitoes often rest in outdoor structures, such as cattle sheds, after feeding on animals, which reduces their exposure to humans and further limits the transmission of the parasite. Combined with low mosquito survival rates, particularly in regions with moderate malaria transmission, this contributes to the observed low sporozoite rates.

These factors, along with others such as incomplete knowledge of mosquito resting sites, highlight the complex dynamics of malaria transmission in Odisha and the need for targeted vector control strategies that consider these behavioural patterns [23–26].

Phylogenetic analysis showed that sibling species S is much different from sibling species T and U, which were closely related (differentiated at only a single position). Similarly, sibling species B/C and A/D were determined at multiple positions, leading to their distinct places in the phylogenetic tree. The percentage of similarity and dissimilarity in different sibling species was due to natural evolution. Host genetic factors also play an influential role in malaria health assessment apart from other clinical and environmental factors [27].

After the successful launch of multiple state [Comprehensive Case Management Programme (CCMP) and the Durgama Anchal are Malaria Nirakarana (DAMaN)] and national [The Global Fund to Fight AIDS, Tuberculosis, and Malaria (GFATM)] vector control programs, various newer interventions have been initiated in the state [28]. Odisha recorded a sharp decline in malaria burden from 2013 to 2018 due to routine health systems, proper surveillance, and improved quality of healthcare and education [29]. Through similar efforts, Odisha can be presented as a role model in a drastic reduction in the number of malaria cases.

We can determine that the low positivity of malaria parasites in *An. culicifacies* screened by ELISA may be because of multiple factors like asymptomatic conditions below the detection level of ELISA and microscopy. A decline in malaria cases was seen in Odisha over the last decade due to various initiatives like integrated intervention, strengthening public health infrastructure, public awareness, and access to medical care.

## Conclusion

Our study confirms that sibling species C of *Anopheles culicifacies* is the primary malaria vector in Rourkela, Sundergarh district, with molecular evidence of *Plasmodium vivax* transmission. This highlights the crucial role of cryptic sibling species in malaria spread, influenced by variations in their ability to transmit the parasite, feeding behavior, and resistance to insecticides. Climate change is further intensifying malaria transmission by shifting vector distribution, increasing breeding sites, and extending seasonal transmission periods. These environmental changes are expanding vector habitats and raising disease risks in endemic regions.

Given the diverse ecological adaptations of sibling species, there is an urgent need for enhanced molecular surveillance and climate-responsive vector control strategies. Species-specific monitoring, genomic tools, and predictive modeling can provide better insights into malaria dynamics, supporting more effective control programs. Future efforts should focus on real-time vector monitoring, community-based interventions, and adaptive control measures. By integrating advanced molecular diagnostics and climate-adaptive strategies, we can strengthen malaria prevention efforts and reduce the disease burden in Sundergarh district and other vulnerable regions.

The impact of climate change and sibling species diversity on malaria transmission necessitates an integrated and dynamic approach to vector surveillance and control. In Sundergarh district, Odisha, ongoing research on malaria vectors must incorporate advanced tools and climate-adaptive strategies to mitigate the disease burden effectively.

## Materials and methods

### Study area:

**Fig. 7:**
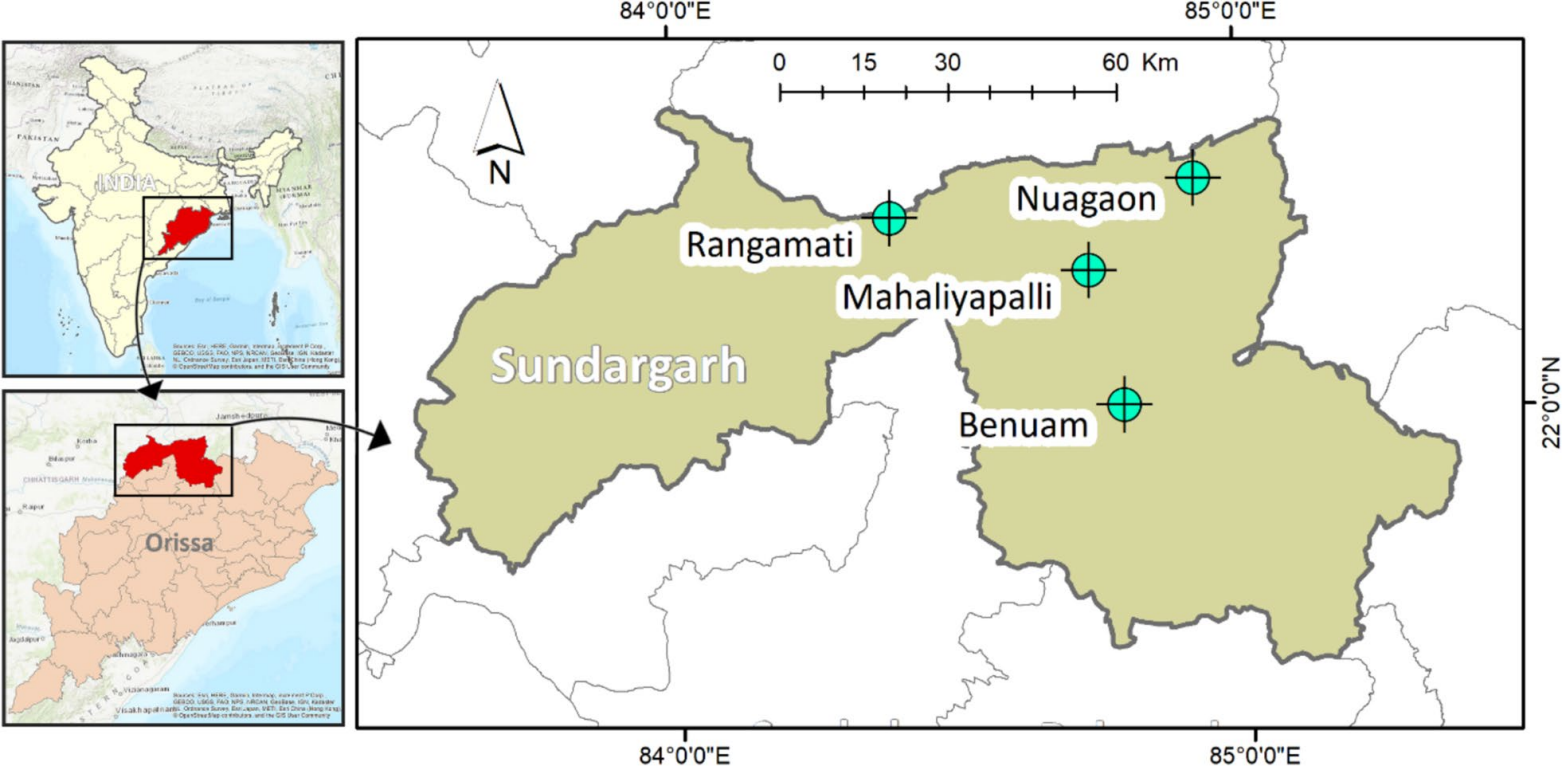
Map of Odisha showing the collection sites/villages (four) from where mosquitoes were collected. Maps were created using QGIS software version 3.14 (https://www.qgis.org/)

Odisha is a hyperendemic region for malaria due to its varied ecotypes. Sundargarh district in Odisha comprises vast, inaccessible forests and extensive river systems that play an essential role in the local vector bionomics. To investigate the local variations in vector prevalence between the two ecosystems, four villages were selected for the study– two located amidst forest ecosystems (Rangamati and Benuam of Gurendia PHC) and two from the riverine flood plains (Mahaliyapalli and Nuagaon of Birkhera PHC). The geographical location of the selected villages is presented in Fig. 7. The study (all experimental protocols) was approved by the NIMR-Institutional Ethical Committee (ECR/65/Inst/DL/2013).

### Generation of climatic, entomological, and fever survey data at field sites of Odisha

Climatic, entomological, and parasitological data were collected in the selected villages for a period of two years from January 2018 to January 2020.

*Generation of climatic data (temperature and rainfall)*: Data on monthly average temperature and total rainfall in all four villages was extracted from the CRU-TS version 4 dataset [16] with the resolution of 0.1^0^ × 0.1^0^. The CRU TS4 dataset compiles climatic data from weather station observations and applies an angular distance weighting technique for interpolating the data.

*Generation of entomological data*: Fortnightly entomological surveys were carried out at selected localities. Data on larval density and adult vector was generated as per standard protocols [30]. Human dwellings and cattle sheds were targeted to collect anopheline mosquitoes using a mouth aspirator and torch between 0600 and 0800 h for 1 h. The man-hour density was calculated from the adult mosquito collections using the formula.

$$Man Hour Density=\frac{Total no. of mosquitoes collected}{No. of persons \times Total time spent in hrs}$$

*Statistical Analysis:* Pearson’s correlation factor was assessed between the climatic and the entomological data collected to identify the impact of climatic factors on vector prevalence.

### Mosquito identification and processing

In total, 4,153 adult mosquitoes were collected. These were morphologically distinguished at the species level using standard keys [31]. After identification, mosquitoes were dried using silica gel to prevent microbial growth and were later used for molecular studies. The NIMR-Institutional Ethical Committee has approved the study.

Dried mosquitoes were dissected into head, thorax, and abdomen parts. The abdominal part of the mosquitoes was used for DNA extraction and identification of sibling species of *An. culicifacies* and *An. fluviatilis* using multiplex PCR. The rest of the body parts (heads and thoraces) were used for the detection of circumsporozoite protein (CSP) of *P. falciparum* (*Pf*) and *P. vivax* (*Pv210* and *Pv247*) using ELISA [32]. Both DNA extraction (using the abdomen) and ELISA (using the head and thorax) were carried out in pools of a maximum of 7 mosquitoes as per the protocol. In the 4,153 adult mosquito samples, the abdomens of 1,765 samples were used for sibling species identification, whereas the head and thorax parts of all 4,153 samples were used for circumsporozoite protein detection through the ELISA test.

### DNA extraction and PCR with universal primers

DNA was extracted using the commercial kit (Qiagen, USA), as per the manufacturer’s protocol. The DNA was stored at -20 °C and later used for conventional PCR. The conserved D3 domain of the 28S rDNA region was amplified using primers D3A: GACCCGTCTTGAAACACGGA and D3B: TCGGAAGGAACCAGCTACTA by PCR [33].

Each PCR was performed with 25.0 μl reaction mixtures in 200 μL PCR tubes. The master mix included: 10 X PCR buffer with MgCl_2_: 2.5 μL, dNTP mix (2.5 mM each): 2.0 μL, forward primer 10 μM: 1.0 μl, reverse primer 10 μM:1.0 μL, template DNA (lysate): 2.5 μL, sterile water: 15.8 μL, Taq polymerase (5 U/μL): 0.2 μL. Amplification was performed at 48 °C for 30 secs. All the reagents were purchased from Qiagen, USA, and amplifications were completed on Bio-Rad thermocycler. PCR products were administered on a 1.5% agarose gel using electrophoresis at 100 V with 15 mA current in an 18-slot apparatus for 30 min and visualized on a gel documentation system. A molecular marker of 100 bp (Promega, India) was used for reference.

### Allele-specific PCR for sibling species

Before the multiplex PCR, sibling species-specific PCR was performed to evaluate the morphologically identified *An. culicifacies* and *An. fluviatilis* using sibling species-specific primers. Later identification of sibling species of both the mosquitoes was done using previously described primers [33, 34]. Amplification was performed at 55 °C for 30 secs, and the rest of the steps were the same as mentioned above.

Morphologically identified mosquitoes were initially amplified using a primer from the D3 region of the 28S rDNA. Further, *An. fluviatilis* specimens were differentiated into S, T, and U sibling species using AFS and AFT primers, while *An. culicifacies* specimens were identified as A/D or B/C/E by D3 PCR. Species belonging to A/D were subjected to ADPCR to differentiate A from D (ADF, ADR, and DF primers), and species belonging to B/C/E are subjected to BCE-multiplex PCR assay (BCR, BCEF, CR, and ER primers) to differentiate between B and C from each other (Table 2).

**Table 2:** Details of primers

S. no	Sequence	Target region
1	GACCCGTCTTGAAACACGGA TCGGAAGGAACCAGCTACTA	D3A D3B
2	TGGAAACCCACAGGCAC TACCCGTAATCCCGCAC	AFS AFT
3	TTAGAGTTTGATTCTTAC	DF
4	CTAATCGATATTTATTACAC TTACTCCTAAAGAAGGC	ADF ADR
5	AAATTATTTGAACAGTATTG TTATTTATTGGTAAAACAAC	BCEF BCR
6	AGGAGTATTAATTTCGTCT GTAAGAATCAAATTCTAAG	CR ER

### ELISA for CSP

Enzyme-linked immunosorbent assays were performed on field-collected mosquitoes using CS-ELISA reagent kit as per the manufacturer’s protocol (*BEI Resources, USA*). A total of 4153 (in 5 batches of 880, 639, 664, 880, and 1090 samples) mosquitoes (3731 cattle shed and 422 house dwellings) including 114 *An. fluviatilis* were screened for the presence of circumsposozoite antigen of *P. falciparum* and *P. vivax*. The kit provided positive control, whereas laboratory-reared mosquitoes were used as the negative control. Mosquito samples were ground and sandwiched between captured monoclonal antibody and peroxidase labeled monoclonal antibody on separate 96 well ELISA plates. ELISA plate results were taken at 405–414 nm using the Microskan spectrum (*Thermo Scientific, USA*). The yellow color's intensity was directly proportional to the amount of CS antigen present in the samples.

### Sequencing

Sequencing was performed commercially (Eurofins, Bangalore, India) for positive PCR products in both directions (forward and reverse) using the same set of primers as mentioned above. The sequences were edited using the Just bio online tool (http://www.justbio.com/hosted-tools.html) and aligned using the multiple sequence alignment program of the Clustal Omega online tool (version 1.2.4). The sequences generated from the study were analyzed using Basic Local Alignment Search Tool (BLAST) and deposited in GenBank database of the National Center for Biotechnology Information (NCBI) and obtained accession numbers.

### Phylogenetic tree

The evolutionary history was inferred by using the Maximum Likelihood method and General Time Reversible model [35]. The bootstrap consensus tree inferred from 100 replicates [36] is taken to represent the evolutionary history of the taxa analyzed. Branches corresponding to partitions reproduced in less than 50% bootstrap replicates are collapsed. The percentage of replicate trees (in which the associated taxa clustered together in the bootstrap test 100 replicates) is shown next to the branches [36].

Initial tree(s) for the heuristic search were obtained automatically by applying Neighbor-Join and BioNJ algorithms to a matrix of pairwise distances estimated using the Maximum Composite Likelihood (MCL) approach and then selecting the topology with superior log likelihood value. A discrete Gamma distribution was used to model evolutionary rate differences among sites (5 categories (+ G, parameter = 0.4874)). The rate variation model allowed for some sites to be evolutionarily invariable ([+ I], 0.00% of sites). This analysis involved 39 nucleotide sequences. Codon positions included were 1st + 2nd + 3rd + Noncoding. There were a total of 1037 positions in the final dataset. Evolutionary analyses were conducted in MEGA11 [37]. 6XU8.A5 is used as an out-group, which is the ribosomal sequence of *Drosophila melanogaster* to construct the tree [38].

## Data Availability

No datasets were generated or analysed during the current study.
